# Detection of suspected cognitive impairment and risk factors among older adults in the Maldives

**DOI:** 10.1590/1980-5764-DN-2026-0560

**Published:** 2026-11-02

**Authors:** Mariyam Fiyaza, Zubair Hassan, Rukhsana Ahmed, Shyh Poh Teo

**Affiliations:** 1 Alzheimer’s Society of Maldives, Malé, Maldives.; 2 Islamic University of Maldives, Malé, Maldives.; 3 Universiti Brunei Darussalam, Pengiran Anak Puteri Rashidah Sa’adatul Bolkiah Institute of Health Sciences, Gadong, Brunei Darussalam.

**Keywords:** Dementia, Cognitive Dysfunction, Risk Factors, Mass Screening, Maldives, Demencia, Disfunción Cognitiva, Factores de Riesgo, Tamizaje Masivo, Maldivas

## Abstract

Dementia is an emerging public health concern in the Maldives due to population aging and increasing noncommunicable diseases.

**Objective:**

To determine the prevalence of self-reported dementia risk factors and symptoms and to estimate the proportion of individuals with suspected cognitive impairment using the Mini-Cog screening tool.

**Methods:**

A cross-sectional, communitybased screening study was conducted in four cities in the Maldives (Male’, Addu, Fuvahmulah, and Kulhudhuffushi). Individuals aged ≥60 years or 50–59 years with at least one dementia risk factor were recruited through community screening. Sociodemographic characteristics, risk factors, cognitive symptoms, and Mini-Cog scores were collected using a structured questionnaire. Suspected cognitive impairment was defined as a Mini-Cog score ≤2.

**Results:**

A total of 940 participants were included (mean age, 62.4 years; 71.2% female). Cardiometabolic risk factors were highly prevalent, including hypercholesterolemia (59.4%), hypertension (50.0%), and diabetes mellitus (36.9%), with over 60% reporting multimorbidity. Common symptoms were memory loss (46.9%) and misplacing items (46.3%); 16.8% reported functional impairment. Among 784 participants with complete Mini-Cog assessments, 278 (35.5%) had suspected cognitive impairment. Multivariable logistic regression showed that increasing age and lower educational attainment were independently associated with suspected cognitive impairment, while hypertension, diabetes mellitus, hypercholesterolemia, and previous stroke were not.

**Conclusion:**

There is a high burden of modifiable risk factors, cognitive symptoms, and suspected cognitive impairment among adults in the Maldives. Community-based screening may facilitate early identification and support dementia risk reduction strategies. Targeted screening of older adults and those with lower educational attainment may improve case detection.

## RESUMEN.

La demencia constituye un problema emergente de salud pública en Maldivas debido al envejecimiento de la población y al aumento de las enfermedades no transmisibles.

**Objetivo:**

Determinar la prevalencia de factores de riesgo y síntomas de demencia autoinformados y estimar la proporción de personas con sospecha de deterioro cognitivo mediante la herramienta de cribado Mini-Cog.

**Métodos:**

Se realizó un estudio de cribado transversal y comunitario en cuatro ciudades de Maldivas (Male’, Addu, Fuvahmulah y Kulhudhuffushi). Se reclutaron personas de ≥60 años o de 50 a 59 años con al menos un factor de riesgo de demencia mediante cribado comunitario. Las características sociodemográficas, los factores de riesgo, los síntomas cognitivos y las puntuaciones del Mini-Cog se recopilaron mediante un cuestionario estructurado. Se definió sospecha de deterioro cognitivo como una puntuación del Mini-Cog ≤2.

**Resultados:**

Se incluyó un total de 940 participantes (edad media: 62,4 años; 71,2% mujeres). Los factores de riesgo cardiometabólicos fueron altamente prevalentes, incluidos la hipercolesterolemia (59,4%), la hipertensión arterial (50,0%) y la diabetes mellitus (36,9%), y más del 60% presentó multimorbilidad. Los síntomas más frecuentes fueron la pérdida de memoria (46,9%) y extraviar objetos (46,3%); el 16,8% reportó deterioro funcional. Entre los 784 participantes con evaluaciones Mini-Cog completas, 278 (35,5%) presentaron sospecha de deterioro cognitivo. La regresión logística multivariable mostró que el aumento de la edad y un menor nivel educativo se asociaron de forma independiente con la sospecha de deterioro cognitivo, mientras que la hipertensión arterial, la diabetes mellitus, la hipercolesterolemia y el antecedente de accidente cerebrovascular no presentaron dicha asociación.

**Conclusión:**

Existe una elevada carga de factores de riesgo modificables, síntomas cognitivos y sospecha de deterioro cognitivo entre los adultos en Maldivas. El cribado comunitario puede facilitar la identificación temprana y apoyar las estrategias de reducción del riesgo de demencia. El cribado dirigido a adultos mayores y a personas con menor nivel educativo podría mejorar la detección de casos.

## INTRODUCTION

 Dementia is a progressive neurocognitive disorder characterized by decline in memory, thinking, and functional abilities sufficient to interfere with daily life. It is a major global public health concern, affecting an estimated 57 million people worldwide, with nearly 10 million new cases each year^1^. As populations age, the burden of dementia is expected to increase substantially, particularly in low- and middle-income countries, where health systems are less equipped to respond. 

 The Maldives is an archipelagic country in South Asia with a small land area of 298 km^2^ dispersed across approximately 1,190 islands, of which 198 are inhabited. Despite demographic and epidemiological transitions, there remains limited data on dementia in the Maldives. Systematic reviews of dementia awareness and neuropsychology research in South Asia have not identified studies conducted in the Maldives, highlighting a significant evidence gap^2,3^. 

 Nevertheless, dementia is an emerging concern in the Maldives. Population projections indicate a rapid increase in the proportion of older adults aged 65 years old and older, from about 4% currently to 14% by 2054^4^. Several modifiable risk factors for dementia are also highly prevalent. The 2020 Lancet Commission on Dementia Prevention, Intervention and Care identified 12 potentially modifiable risk factors accounting for 40% of dementia globally^5^. In the Maldives, the STEPwise approach to noncommunicable diseases (STEPS) survey found a high prevalence of cardiometabolic risk factors, including hypertension in one-third of the population and central obesity and hyperlipidemia in more than half^6^. Furthermore, estimates from the Global Burden of Disease Study 2019 suggest that the number of people living with dementia in the Maldives will increase markedly, from 1,703 cases in 2019 to 11,135 people in 2050^7^. These trends indicate a need for improved detection, risk reduction strategies, and the development of health and social care systems to support people living with dementia. 

 Dementia prevention involves primary prevention to prevent people with normal cognitive function from developing dementia, secondary prevention focusing on the early detection and treatment of dementia, and tertiary prevention involving the management and slowing of dementia progression^8^. For the Maldives, improving public awareness of brain health and dementia risk factors, alongside opportunistic case-finding, may facilitate earlier identification and intervention. A study from Belgium found that awareness of dementia risk reduction was limited, with a majority indicating that they would welcome more information on improving brain health^9^. 

 Community-based screening has been proposed as a pragmatic approach to improving awareness and early identification of cognitive impairment^10^. In Kenya, community health worker-led screening was feasible and involved asking basic questions about risk factors and administering basic cognitive testing^11^. The Mini-Cog is a brief and practical cognitive screening tool that combines a three-item recall task and a clock-drawing test. It has demonstrated good sensitivity for detecting cognitive impairment^12^, performs comparably to other screening tools^13^, and has been applied in diverse non-English-speaking settings, including Kashmir and Eastern China^14,15^. The Mini-Cog was selected for this study because it requires minimal training and can be administered in approximately three minutes^12,13^, making it particularly suitable for opportunistic community-based screening in geographically dispersed island nations such as the Maldives, where access to specialist cognitive assessment may be limited. 

 There is a need for locally relevant data to better characterize dementia risk factors, symptoms, and suspected cognitive impairment in the Maldives. This study aimed to conduct community-based screening in four cities in the Maldives to determine the prevalence of self-reported dementia risk factors and symptoms and estimate the proportion of individuals with suspected cognitive impairment using the Mini-Cog. 

## METHODS

 This was a cross-sectional, community-based screening study conducted in four cities in the Maldives: Male’, Addu, Fuvahmulah, and Kulhudhuffushi. Data collection was carried out between October 2024 and May 2025. 

 Participants were recruited through voluntary community-based screening. Individuals were eligible if they were aged 60 years old and older or aged 50-59 years with at least one self-reported dementia risk factor, such as hypertension, diabetes mellitus, cardiovascular disease, renal impairment, or previous stroke. Participants were residents of the selected cities, including both Maldivians and foreign residents. Individuals who did not meet the inclusion criteria were excluded from the analysis. Participants who required assistance due to literacy or language barriers were supported by trained volunteers or accompanying family members. Responses regarding cognitive symptoms were obtained primarily from participants themselves. Accompanying family members assisted only with clarification or translation where required and were not routinely used as collateral informants. 

 Data were collected using a structured questionnaire administered in person by trained volunteers. Screening was conducted in accessible community venues, including schools, healthcare facilities, public events, and areas where older people socialize. The questionnaire consisted of five components: sociodemographic information (age, sex, education level, and occupation); self-reported dementia risk factors; symptoms of possible cognitive impairment; red flag indicators, such as worsening symptoms, functional impact, and perceived need for assessment; and the Mini-Cog. The questionnaire was available in English and Dhivehi and was pilot tested among older adults to assess clarity and comprehension prior to data collection. 

 Scoring the Mini-Cog involved adding the scores for the three-item recall (maximum score, 3) and the clock-drawing test (maximum score, 2). Participants with a total Mini-Cog score of 2 or below were classified as having suspected cognitive impairment. Participants with incomplete Mini-Cog assessments were excluded from analyses involving cognitive outcomes. 

 Participant data were anonymized using unique identifiers prior to analysis. Data were entered into Microsoft Excel and analyzed using IBM SPSS Statistics for Windows, Version 22.0 (IBM Corp., Armonk, NY, USA). Continuous variables were summarized using means and standard deviations (SD), while categorical variables were presented as frequencies and percentages. The prevalence of self-reported risk factors and symptoms was calculated. The proportion of participants with suspected cognitive impairment was determined among those with complete Mini-Cog data. Data were reviewed for completeness and consistency prior to analysis. Associations between participant characteristics and suspected cognitive impairment were initially examined using Pearson’s chi-square tests. Variables considered clinically relevant were subsequently entered into a multivariable logistic regression model using complete-case analysis to identify factors independently associated with suspected cognitive impairment. Crude and adjusted odds ratios (OR) with 95% confidence intervals (95%CI) were calculated. Statistical significance was defined as p<0.05. 

 Ethical approval was obtained from the National Health Research Council, Ministry of Health, Maldives (NHRC/2024/68). Participation was voluntary, and written informed consent was obtained from all participants. Participants identified with potential cognitive impairment were advised to seek further medical evaluation through health facilities with neurologists, which is the currently used pathway for persons with suspected dementia. 

## RESULTS

### Participant enrollment

 A total of 1,073 individuals were screened (Figure 1). Of these, 940 (87.6%) individuals met the inclusion criteria and were included in the analysis, while 133 (12.4%) were excluded. Inclusion rates varied by city, ranging from 77.6% in Addu to 96.5% in Kulhudhuffushi. The largest proportion of participants was recruited from Male’ (n=543, 57.8%), followed by Addu (n=215, 22.9%), Kulhudhuffushi (n=110, 11.7%), and Fuvahmulah (n=72, 7.7%). Across all cities, 400 participants (42.6%) were aged 50–59 years, and 540 (57.4%) were aged ≥60 years. 

**Figure 1:**
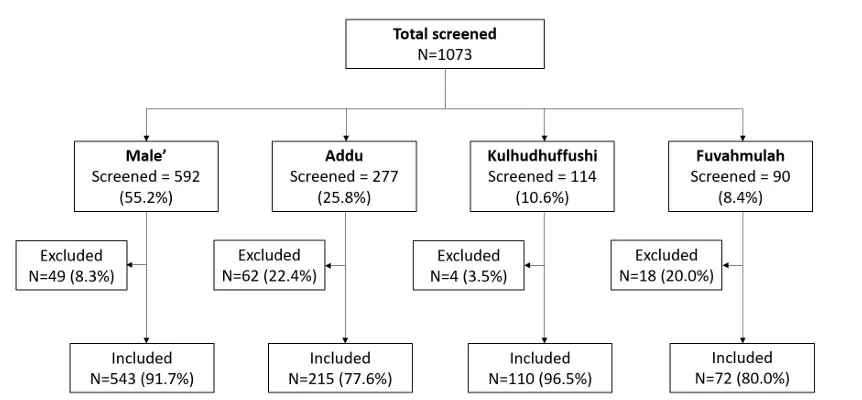
Flow diagram of participant screening and inclusion across four cities in the Maldives.

### Demographic characteristics

 The mean age of participants was 62.4 years (SD, 8.5), with similar age distributions across cities. The largest age groups were 50-59 years (42.6%) and 60-69 years (39.3%) (Table 1). Females constituted the majority of participants (71.2%), with a similar pattern observed across cities. Education levels varied across the cities. Primary education was the most common level overall (34.3%), while a significant proportion reported non-formal education (40.9%). Higher levels of secondary and tertiary education were more common in Male’ compared with other cities. Kulhudhuffushi and Fuvahmulah had a high proportion of participants with non-formal educational pathways, such as *Assassee Thauleem*, a locally recognized form of community-based education outside the formal school system. 

**Table 1:** Participant characteristics by city

Variable		Total (n=940)	Male’ (n=543)	Addu (n=215)	Kulhudhuffushi (n=110)	Fuvahmulah (n=72)
Age group						
	50-59 years	400 (42.6)	227 (41.8)	112 (52.1)	41 (37.3)	20 (27.8)
	≥60 years	540 (57.4)	316 (58.2)	103 (47.9)	69 (62.7)	52 (72.2)
Sex						
	Female	669 (71.2)	397 (73.1)	154 (71.6)	73 (66.4)	45 (62.5)
	Male	271 (28.8)	146 (26.9)	61 (28.4)	37 (33.6)	27 (37.5)
Education						
	None	18 (1.9)	16 (2.9)	2 (0.9)	0	0
	Primary	322 (34.3)	188 (34.6)	97 (45.1)	27 (24.5)	10 (13.9)
	Secondary	107 (11.4)	86 (15.8)	16 (7.4)	3 (2.7)	2 (2.8)
	Tertiary	53 (5.6)	43 (7.9)	6 (2.8)	3 (2.7)	1 (1.4)
	Non-formal	384 (40.9)	194 (35.7)	81 (37.7)	66 (60.0)	43 (59.7)
	Undocumented	56 (6.0)	16 (2.9)	13 (6.0)	11 (10.0)	16 (22.2)

### Modifiable risk factors

 Cardiometabolic risk factors were highly prevalent in the study population (Table 2). The most commonly reported conditions were hypercholesterolemia (59.4%), hypertension (50.0%), and diabetes mellitus (36.9%). Regarding lifestyle factors, 35.4% of participants reported being overweight. Current smoking was reported by 15.9%, while 22.3% identified as former smokers. Other significant conditions included previous head injury (22.1%), heart disease (11.9%), and stroke (9.0%). The proportion of missing or "don’t know" responses was low across most variables. 

**Table 2:** Prevalence of self-reported dementia risk factors (n=940).

Risk factor	Yes, n(%)	No, n(%)	Don’t know, n(%)	Undocumented, n(%)
Hypertension	470 (50.0)	456 (48.5)	13 (1.4)	1 (0.1)
Hypercholesterolemia	558 (59.4)	366 (38.9)	16 (1.7)	
Diabetes mellitus	347 (36.9)	583 (62.0)	8 (0.9)	2 (0.2)
Heart disease	112 (11.9)	775 (82.4)	44 (4.7)	9 (1.0)
Lung disease	116 (12.3)	786 (83.6)	37 (3.9)	1 (1.0)
Kidney disease	54 (5.7)	844 (89.8)	38 (4.0)	4 (0.4)
Liver disease	36 (3.8)	863 (91.8)	39 (4.1)	2 (0.2)
Previous head injury	208 (22.1)	696 (74)	31 (3.3)	5 (0.5)
Previous stroke	85 (9.0)	836 (88.9)	14 (1.5)	5 (0.5)
Overweight	333 (35.4)	570 (60.6)	19 (2.0)	18 (1.9)
Current smoker	149 (15.9)	569 (60.5)	-	12 (1.3)
Former smoker	210 (22.3)	-	-	-

### Multimorbidity of cardiometabolic conditions

 Multimorbidity was common in the study population. The most frequent combination of risk factors was the lesterolemia, and diabetes in 24.3% of participants. Dual combinations were also prevalent, including hypertension with hypercholesterolemia (23.8%) and hypercholesterolemia with diabetes (10.2%). In contrast, single-condition profiles were less common. Overall, more than 60% of participants had two or more cardiometabolic conditions, indicating a substantial burden of multimorbidity. 

### Dementia-related symptoms and functional features

 The prevalence of dementia-related symptoms is shown in Table 3. The most commonly reported symptoms were memory loss (46.9%), misplacing items (46.3%), and mood or behavioral changes (24.8%). A total of 184 participants (19.6%) thought their cognitive symptoms were worsening, 158 (16.8%) indicated that symptoms affected activities of daily living, and 611 (65.0%) thought they should have their cognition checked. 

**Table 3:** Prevalence of dementia-related symptoms (n=940).

Symptom / Indicator		Yes, n(%)	No, n(%)	Undocumented, n(%)
Cognitive symptoms				
	Memory loss	441 (46.9)	495 (52.7)	4 (0.4)
	Misplacing items	435 (46.3)	501 (53.3)	4 (0.4)
	Mood or behavioral changes	233 (24.8)	702 (74.7)	5 (0.5)
	Difficulty performing tasks	212 (22.6)	727 (77.3)	1 (0.1)
	Language difficulties	206 (21.9)	731 (77.8)	3 (0.3)
	Judgement problems	195 (20.7)	740 (78.7)	3 (0.3)
	Visuospatial difficulties	176 (18.7)	758 (80.6)	6 (0.6)
	Difficulty keeping track of things	136 (14.5)	795 (84.6)	9 (1.0)
	Withdrawing from work or social activities	133 (14.1)	804 (85.5)	3 (0.3)
	Orientation difficulties	127 (13.5)	810 (86.2)	3 (0.3)

### Cognitive screening outcomes

 Of the 940 participants, 784 had complete Mini-Cog assessments. Among these, 278 (35.5%) were classified as having suspected cognitive impairment based on the Mini-Cog scores, while 506 (64.5%) were classified as not impaired. A total of 156 participants (16.6%) had incomplete assessments and were excluded from the cognitive outcome analysis. 

### Factors associated with suspected cognitive impairment

 Factors associated with suspected cognitive impairment are shown in Table 4. In univariate analyses, increasing age, lower educational attainment, and previous stroke were significantly associated with suspected cognitive impairment. Following multivariable adjustment, increasing age and lower educational attainment remained independently associated with suspected cognitive impairment, whereas hypertension, diabetes mellitus, hypercholesterolemia, previous stroke, previous head injury, and current smoking were not independently associated. 

**Table 4:** Univariate and multivariable logistic regression analyses of factors associated with suspected cognitive impairment among participants with complete Mini-Cog assessments (n=784).

Variable	Crude OR (95%CI)	p-value	Adjusted OR (95%CI)	p-value
Age 60–69 years	1.92 (1.39–2.65)	<0.001	1.85 (1.29–2.66)	0.001
Age 70–79 years	1.92 (1.18–3.12)	0.008	1.85 (1.05–3.25)	0.034
Age ≥80 years	7.27 (2.76–19.10)	<0.001	8.51 (2.57–28.13)	<0.001
Male sex	1.03 (0.75–1.42)	0.850	0.92 (0.62–1.37)	0.669
Primary education	2.11 (1.31–3.39)	0.002	1.71 (1.03–2.82)	0.037
None/non-formal education	3.07 (1.93–4.88)	<0.001	2.68 (1.64–4.38)	<0.001
Hypertension	1.20 (0.89–1.61)	0.234	0.89 (0.62–1.28)	0.537
Diabetes mellitus	0.96 (0.71–1.31)	0.797	0.98 (0.69–1.39)	0.910
Hypercholesterolemia	1.18 (0.87–1.60)	0.275	1.29 (0.90–1.84)	0.173
Previous stroke	1.74 (1.04–2.90)	0.034	1.24 (0.70–2.21)	0.464
Previous head injury	1.25 (0.88–1.76)	0.217	1.22 (0.82–1.81)	0.321
Current smoker	1.15 (0.78–1.69)	0.481	1.15 (0.73–1.83)	0.543

Abbreviations: OR, odds ratio; CI, confidence interval. Notes: Reference categories were age 50–59 years, female sex, secondary/tertiary education, absence of hypertension, diabetes mellitus, hypercholesterolemia, previous stroke, previous head injury, and current smoking.

## DISCUSSION

 This study provides new insights into the distribution of dementia risk factors, cognitive symptoms, and suspected cognitive impairment among adults aged 50 years old and older in the Maldives. To our knowledge, this study represents the first large community-based cognitive screening study conducted in the Maldives and provides important baseline epidemiological data for a country where evidence on dementia remains scarce. The findings identified a population with a high burden of cardiometabolic risk factors and early cognitive vulnerability, indicating a need for integrated dementia prevention and early detection strategies. 

 The demographic profile reflects a relatively younger at-risk population, with a significant proportion of participants aged 50-59 years included due to the presence of risk factors. This approach is consistent with a prevention-oriented strategy targeting midlife risk, which is increasingly recognized as a critical window for dementia risk reduction^5^. The predominance of female participants is consistent with patterns observed in community-based studies, where women are more likely to participate in health-related activities^16^. Educational disparities, including a high proportion of individuals with primary or non-formal education, are relevant given the established relationship between lower educational attainment and reduced cognitive reserve^5,17^. 

 An important finding from this study was that increasing age and lower educational attainment were independently associated with suspected cognitive impairment. Cardiometabolic multimorbidity was also highly prevalent, with more than half of the participants reporting two or more conditions, and nearly a quarter having the combination of hypertension, hypercholesterolemia, and diabetes mellitus. These conditions have consistently been identified as important modifiable risk factors for dementia and are strongly associated with both vascular cognitive impairment and Alzheimer disease. The clustering of these conditions within individuals suggests a compounded risk profile^18,19^, supporting integrated management strategies that address multiple risk factors simultaneously to reduce dementia risk in the Maldives. Although hypertension, diabetes mellitus, hypercholesterolemia, and previous stroke were not independently associated with suspected cognitive impairment in this cross-sectional analysis, these conditions remain well-established modifiable dementia risk factors identified in longitudinal cohort studies^5^. 

 Lifestyle-related factors also contribute to this risk profile. Approximately one-third of participants were overweight, with a similar proportion with tobacco exposure, which increases vascular risk. These patterns are consistent with epidemiological trends in South Asia, where rapid urbanization and lifestyle transitions contribute to the increasing prevalence of noncommunicable diseases^20,21^. The convergence of cardiometabolic and lifestyle-related risk factors underscores the need for greater emphasis on comprehensive population-level interventions focusing on health promotion, behavioral modification, and risk factor control. 

 There was a high prevalence of self-reported cognitive symptoms, particularly memory loss, misplacing items, and mood or behavioral changes. A total of 16.8% of participants reported functional impact on activities of daily living, suggesting clinically significant impairment^22^. While a significant proportion felt they needed to have their cognition checked, gaps remain between symptom recognition and help-seeking behavior, indicating the need for continued public education and awareness^20,23^. 

 The proportion of participants classified as having suspected cognitive impairment based on the Mini-Cog (35.5%) was higher than expected. This may be due to the targeted inclusion of individuals with risk factors, as well as the opportunistic nature of community-based recruitment. Rather than representing population prevalence, these findings illustrate the potential of screening initiatives to identify individuals at increased risk who may otherwise remain undiagnosed. Early identification in such populations provides an opportunity for timely intervention, risk factor optimization, and further clinical evaluation. 

 In a community-based dementia screening program conducted in Brunei, a lower proportion of participants (14.4%) were classified as having suspected cognitive impairment^24^. This difference may reflect variations in study design, participant selection, and screening contexts. The present study included younger individuals with risk factors and relied on voluntary participation, which may enrich the sample for individuals with symptoms or concerns. These findings highlight the importance of considering the screening context when interpreting prevalence estimates and making comparisons across settings. 

 This study also supports the feasibility of delivering community-based cognitive screening across diverse settings. Screening was conducted in public, community, and semi-clinical environments, demonstrating that opportunistic case-finding can be integrated into routine public health and community activities. Screening programs can also be effectively delivered by trained non-specialist personnel, including students and volunteers, under appropriate supervision^25^. This task-sharing approach is particularly relevant for the Maldives, where specialist resources may be limited, and provides a scalable model for expanding early detection efforts. 

 This study has several strengths, including a large sample size, inclusion of participants across multiple community settings, and the use of a brief, pragmatic screening approach that is feasible in real-world contexts. However, there are several limitations. The cross-sectional design precludes causal inference or assessment of progression. Risk factors and symptoms were self-reported and may be subject to recall bias or underreporting. Furthermore, cognitive symptoms were obtained primarily from participants rather than collateral informants. Reduced insight (anosognosia) among individuals with cognitive impairment may therefore have resulted in underestimation of subjective cognitive symptoms. The purposive sampling approach may limit generalizability, particularly for individuals who are homebound or less likely to participate in community activities. In addition, the Mini-Cog is a screening tool and does not provide a definitive diagnosis of cognitive impairment or dementia. 

 Future research should consider longitudinal follow-up of screened individuals to determine the predictive value of cognitive screening and symptom reporting for subsequent diagnosis. Validation studies comparing the Mini-Cog with more comprehensive cognitive assessment instruments, such as the Montreal Cognitive Assessment (MoCA), the Rowland Universal Dementia Assessment Scale (RUDAS), or formal neuropsychological assessment within the Maldivian population, should be undertaken to determine the most appropriate cognitive screening strategy for the Maldivian population. There is also a need to develop culturally appropriate intervention strategies, including lifestyle modification programs, cognitive stimulation, and caregiver support. Comparative studies across similar settings may further elucidate how differences in education, healthcare access, and social context influence dementia risk and detection. 

 In conclusion, this study found a high burden of modifiable risk factors, cognitive symptoms, and suspected cognitive impairment among adults in the Maldives. Increasing age and lower educational attainment were independently associated with suspected cognitive impairment. The findings support the implementation of community-based, opportunistic screening strategies integrated with public health initiatives, alongside strengthened efforts in risk reduction and early intervention to mitigate the future burden of dementia. 

## Data Availability

The datasets generated and analyzed during the current study are available from the corresponding author upon reasonable request.
